# CellDestiny: A RShiny application for the visualization and analysis of single-cell lineage tracing data

**DOI:** 10.3389/fmed.2022.919345

**Published:** 2022-10-05

**Authors:** Louisa Hadj Abed, Tamar Tak, Jason Cosgrove, Leïla Perié

**Affiliations:** ^1^Institut Curie, Université PSL, Sorbonne Université, CNRS UMR168, Laboratoire Physico Chimie Curie, Paris, France; ^2^Centre de Bio-Informatique, MINES ParisTech, Institut Curie, PSL University, Paris, France

**Keywords:** lineage tracing, single-cell, bioinformatics, gene therapy, data analysis, lentiviral barcoding

## Abstract

Single-cell lineage tracing permits the labeling of individual cells with a heritable marker to follow the fate of each cell’s progeny. Over the last twenty years, several single-cell lineage tracing methods have emerged, enabling major discoveries in developmental biology, oncology and gene therapies. Analytical tools are needed to draw meaningful conclusions from lineage tracing measurements, which are characterized by high variability, sparsity and technical noise. However, the single cell lineage tracing field lacks versatile and easy-to-use tools for standardized and reproducible analyses, in particular tools accessible to biologists. Here we present CellDestiny, a RShiny app and associated web application developed for experimentalists without coding skills to perform visualization and analysis of single cell lineage-tracing datasets through a graphical user interface. We demonstrate the functionality of CellDestiny through the analysis of (i) lentiviral barcoding datasets of murine hematopoietic progenitors; (ii) published integration site data from Wiskott-Aldrich Symdrome patients undergoing gene-therapy treatment; and (iii) simultaneous barcoding and transcriptomic analysis of murine hematopoietic progenitor differentiation *in vitro*. In summary, CellDestiny is an easy-to-use and versatile toolkit that enables biologists to visualize and analyze single-cell lineage tracing data.

## Introduction

Single-cell lineage tracing allows the labeling of single cells with a heritable marker to follow the fate of their progeny (1, 2). Heritable markers can be introduced in a variety of ways (2), most commonly by using lentiviruses to introduce a synthetic genetic sequence (barcode) into the genomic DNA of infected cells in an approach known as cellular barcoding (3–6). Other labeling methods include integration site analysis, where cells are also infected with a lentivirus, but the heritable marker is based on the genomic location of the viral integration rather than a unique nucleotide sequence (7). Virus-free labeling methods include the use of CRISPR-cas9 gene-editing (8–11), as well as the Cre-Lox (12) and transposon (13) genetic technologies. Virus-free *in situ* lineage-tracing technologies are advantageous because cells are barcoded within a living organism without the need to remove a cell from its microenvironment, but these methods are restricted to genetically engineered animals. To overcome this limitation, retrospective lineage-tracing approaches have emerged for human samples (14). This is made possible by analyzing naturally-occurring genetic variations that accumulate over time, for example single nucleotide polymorphisms (SNPs) (15, 16).

Single cell lineage-tracing approaches have yielded significant insights into the developmental history of normal, cancerous, and gene-edited cells *in vivo* (14). A key area of study where lineage-tracing has been applied is hematopoiesis (17), the process by which new blood and immune cells are produced in the bone marrow. In this context, the contribution of single cell lineage-tracing ranges from revealing significant heterogeneity in apparently homogenous cell intermediates (18, 19), to stimulating revisions to the topology of the tree in steady state and upon emergency hematopoiesis [Perié et al. (20); Rodriguez-Fraticelli et al. (21); Eisele et al. (22); Wu et al. (23); Lin et al. (24)], to comparing the dynamics of naïve versus post-transplantation hematopoiesis (13, 25–27), to identifying molecular regulators of cell fate (8, 28, 29). In a clinical context, single cell lineage tracing has revealed how cellular dynamics correlate with treatment efficacy and as well as assessing the potential for vector genotoxicity (6, 30).

Despite the seminal contribution of single-cell lineage-tracing, the field lacks versatile and easy-to-use tools for standardized and reproducible analyses. This point is critical, because the analysis of lineage tracing datasets is non-trivial, with data characterized by high variability, sparsity and technical noise. Complex analysis pipelines have been developed to tackle these challenges, but they require significant expertise in both computer programming and statistics to implement and evaluate, precluding their usage by biologists who cannot code. This is particularly limiting for the biological interpretation of the data in which the biologists play an active role together with bioinformaticians. There is therefore a need for tools that permit the exploration of lineage tracing datasets, without needing to write computer code. Increasing the availability of open-source tools will make lineage-tracing analysis more reproducible, transparent, and accessible—as well as permitting meta-analyses across independent studies (31, 32).

**FIGURE 1:**
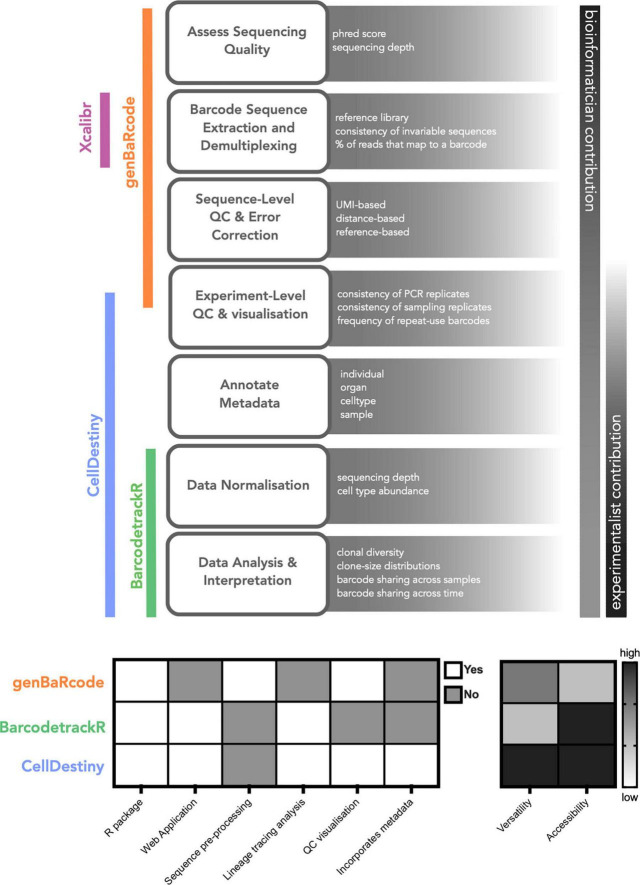
Computational tools for lineage tracing data analysis. A generalized scheme for lineage tracing data analysis highlighting the relative contribution of experimentalists and analysts as well as the associated analysis toolkits for each step. We also provide a qualitative comparison of the different toolkits to illustrate their key functionality. When comparing the utilty of different toolkits the term versatility refers to whether the tool can incorporate metadata into the analysis framework, enabling comparisons across and within technical and biological replicates.

Currently available software tools to perform lineage-tracing analysis can be divided into two key categories: (1) Preprocessing toolkits that perform recovery, filtering and quality control of genetic sequences from raw sequencing reads and (2) Analysis toolkits that perform visualization and analysis of barcode abundances across biological samples (Figure 1). Pre-processing toolkits are typically context-specific, in that the retrieval, QC and quantification of genetic sequences will vary depending on the nature of the barcode. The open-source toolkit genBaRcode has been developed to address this issue, performing sequence retrieval and processing across a range of different genetic labeling strategies, as well as some data visualization tools (33). Xcalibr is also a pre-processing toolkit, that focuses only on the extraction and demultiplexing of barcodes from sequencing data (18, 19). With regards to analysis toolkits, barcodetrackR has been developed for the analysis of clonal tracking data over time (31) (Figure 1). BarcodetrackR is a versatile tool that places an emphasis on longitudinal analyses. However it does not permit the visualization of key QC metrics, such as the consistency of measurements across technical replicates, which are an important consideration when drawing biological conclusions from the data. In addition, for parameters other than time, such as treatments or organ localization, the visualization of the data is limited.

Here, we present a new complementary analysis toolkit called CellDestiny, enabling the visualization and analysis of single-cell lineage tracing data through an easy-to-use web application. CellDestiny is an easy to use app that facilitates data exploration and extraction of biological insights from single cell lineage tracing data without needing to write computer code. We envision that CellDestiny will enrich collaborations between biologists and bioinformaticians without replacing the crucial work of the latter. The key advantages of CellDestiny are: (i) the ability to incorporate study metadata into the analysis framework, (ii) the focus on experiment-level quality control visualizations that can be used to assess the impact of technical factors like sequencing errors and repeat-used barcodes (34), and (iii) the visualization of data using a wide range of plotting methods (Figure 1). Compared to other toolkits, CellDestiny provides a greater flexibility in data plotting, in that it allows the user to choose which data to visualize using several grouping options, for example across individuals, organs and cell types. To demonstrate the functionality and versatility of CellDestiny, we analyze data from three different lineage-tracing studies, including lentiviral barcoding analysis of murine hematopoietic progenitor differentiation *in vivo* (previously unpublished), lentiviral integration site analysis of patients undergoing gene-therapy (30) and simultaneous barcoding and transcriptomic analysis of murine hematopoietic progenitor differentiation *in vitro* (35).

## Results

### Overview of CellDestiny

CellDestiny is a data analysis tool developed for lineage-tracing experimentalists without coding skills. Focusing on this user group is important as nuanced experimental details can influence the interpretation of the results, and so experimentalists need to play an active role in the analysis. The purpose of this app is to provide users who cannot write computer code with greater autonomy to analyze, explore and extract meaningful information from lineage tracing datasets. Cell Destiny is available as a Rshiny app or web application. The R package associated with CellDestiny is a collection of plotting and analysis tools available to experimentalists who prefer to write their own code rather than using the graphical user interface.

CellDestiny requires two key inputs: (1) a count matrix that has undergone sequence and experiment level quality control (QC) preprocessing where each row (*x*) is a unique barcode sequence and each column (*y*) is a sample. The numeric value for each *x,y* pairing then represents the number of times the barcode label *x* was measured in sample *y.* (2) A metadata table providing information about each sample which may include the experimental timepoint, cell type and individual identity. Additional information such as pre-calculated QC metrics and information about experimental design such as sequencing batch can also be uploaded through the metadata feature and overlaid onto QC and analysis plots.

**FIGURE 2:**
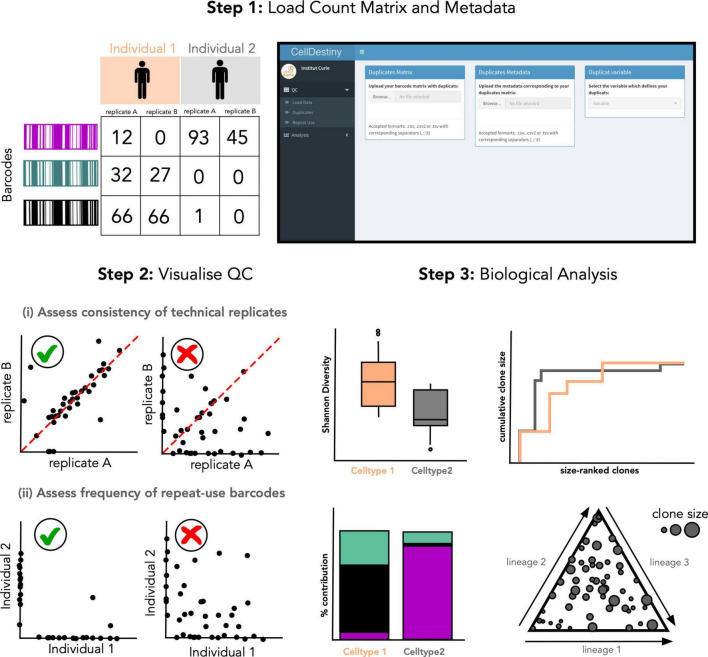
Overview of the CellDestiny analysis toolkit. (Step 1) A count matrix containing technical replicates for each sample after data quality control and normalization steps, for example using the genBaRcode package, can be uploaded along with associated metadata detailing the variables used for the analysis such as organ, time point, individual etc. (Step 2) The quality of the data can be visualized to assess the correlation between duplicates and also the frequency of repeat-used barcodes. These steps will help the user to assess the impact of technical artifacts on the biological interpretation of the data. (Step 3) The data summed or averaged over replicates can then be uploaded and visualized through a set of different graphs to assess clonal diversity, clone-size distribution and fate outcomes. Importantly, these graphs offer several customization options such as grouping data across individuals, cell types and organs.

In the CellDestiny workflow (Figure 2), the user can first visualize QC from pre-processed data for each sample independently (see next section) before performing analyses such as the quantification of barcode sharing, clone size, barcode diversity and lineage biases across individuals and samples. CellDestiny allows the user to choose which data to visualize using several grouping options. As the grouping option depends on the biological question being addressed by the experiment, CellDestiny has been designed to allow greater flexibility in data exploration than previous tools. Each graph generated can be exported as an image, as well the associated reformatted matrix to be uploaded for statistical analysis with external software packages such as Prism. Cell Destiny is not tailored for statistical analysis. A graphical overview of the package is provided in Figure 1, and a table comparing CellDestiny to the complementary barcodeTrackR toolkit is provided in Supplementary Table 1.

### Quality controls

Quality control (QC) is an important part of the analysis of single-cell lineage tracing data but there is currently no clear consensus on how data QC should be done. Different guidelines have been published (34, 36) and while some software packages exist to perform pre-processing of genetic sequences (33) many pipelines are custom-built for each distinct barcode design. It is therefore challenging to create a fully automated QC pipeline that is applicable across all single-cell lineage tracing technologies. While we advocate that data QC should be done by bioinformatic experts, we believe that QC data be made accessible to biologists with no coding skills, such that they can better draw biological interpretations from the data.

Quality control steps for lineage tracing experiments can be subdivided into two key sub steps: (i) sequence level QC assesses the genetic sequences encoding heritable markers and (ii) experiment-level QC that assesses the consistency of results within and between biological and technical replicates (Figure 1). To this end, CellDestiny is designed to allow experiment-level QC visualization (Figure 2). There are two important experiment-level QC steps before proceeding to analysis of barcoding data, (1) the correlation between PCR technical replicates and (2) the evaluation of repeat usage of barcodes (34). Firstly, PCR technical replicates allow to measure the degree of confidence in barcode detection to establish lineage relationship between cell types. If the replicates do not show high correlation, there may an issue with barcode contamination or it may suggest poor recovery of daughter cells or poor expansion of progenitors. In CellDestiny, technical replicates can be uploaded and visualized for each sample. Secondly, the integration of the same barcode into multiple cells, called repeat usage, is also an important QC metric that should be considered in a lineage tracing analysis pipeline, as a high incidence of repeat usage may lead to false lineage relationship assignments. The transfer of progenitors from the same transduction batch into at least two separate mice, followed by subsequent comparison of the barcodes recovered from those mice, can be used to estimate the frequency of repeat barcode use within one mouse. In CellDestiny, this QC can be visualized with 2D plot of barcode sharing between two mice or with a heatmap when analyzing sharing across more than 2 mice (Figures 2A, 4). It is important to state that PCR/sequencing errors and multiple integrations are not the only source of confounding factors in lineage tracing experiments. Insufficient cell sampling can lead to poor barcode recovery and greatly influence the interpretation of the data and so must be considered when interpreting the results. Where available several tissue sampling replicates can be compared in CellDestiny through the metadata functionality.

In the following sections we demonstrate the functionality of CellDestiny through 3 case studies: (1) lentiviral barcoding of murine hematopoietic progenitors (2) lentiviral hematopoietic stem cell gene therapy in patients with Wiskott-Aldrich Syndrome (3) lentivirus barcoding and transcriptomic analysis of murine hematopoietic progenitors, cultured in vitro. A table summarizing each dataset, along with key metadata and information about how to access the raw data is provided in Supplementary Table 2.

## Case study 1: Lentiviral barcoding of murine hematopoietic progenitors

### Study overview

In case study 1, we will study the development of lung-resident conventional dendritic cells in mice. Dendritic cells are a key component of the immune system, initiating adaptive immune responses through the presentation of antigen (37). DCs (CD11c^+^MHCII^+^CD24^–^CD64) can be further divided into a number of sub-categories including the conventional cDC1 and cDC2. cDC1, identified by their surface expression of CD8α, present antigens and prime cytotoxic CD8^+^ T cell responses to intracellular pathogens (37). cDC2, defined by their surface expression of the CD11b marker, constitute a more heterogeneous cell population that preferentially activate CD4^+^ helper T cell responses (38).

**FIGURE 3:**
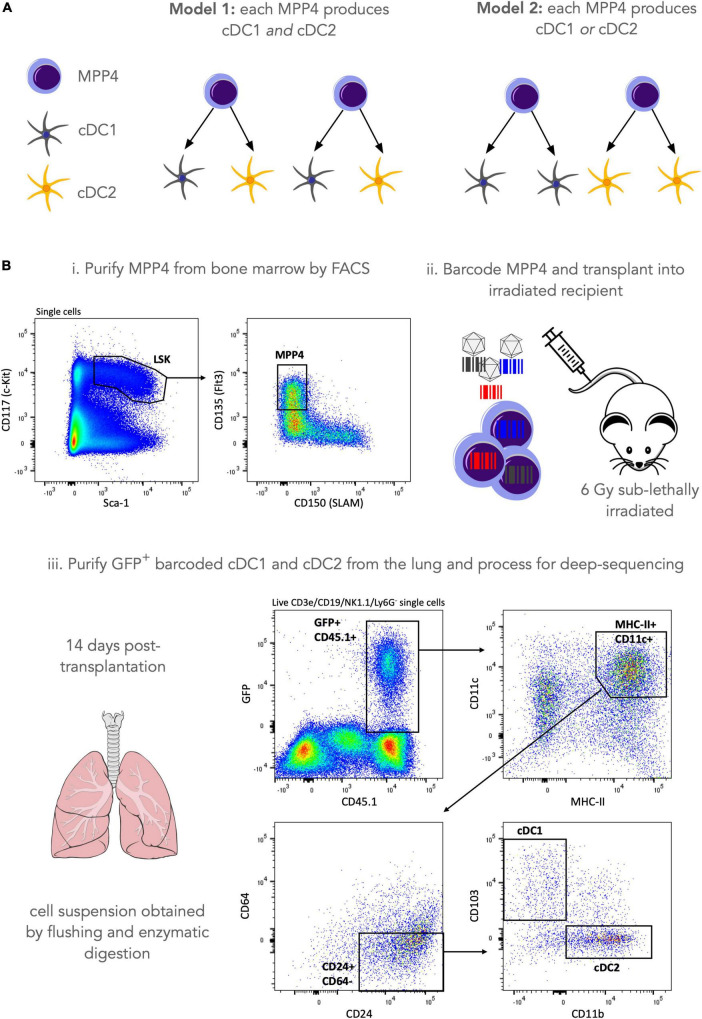
Lentiviral barcoding of murine hematopoietic progenitors. **(A)** Our research question is to assess whether a single MPP4 can make both cDC1 and cDC2 lung-resident populations (model 1) or whether they are fate restricted to produce only one of the two cell types (model 2). **(B)** To address this research question, we purified MPP4 by FACS sorting and labeled cells with a lentiviral barcoding library. Cells were then transplanted into 3 irradiated (6Gy sub-lethal) recipients and left to engraft, divide and differentiate for 14 days. 14 days post-transplantation lungs were harvested and GFP+ (barcode-expressing) cDC1 and cDC2 were purified by FACS sorting, and their genomic DNA was processed for sequencing library preparation as described in the materials and methods section. After sample QC a total of 60 barcodes were recovered for lung cDCs across 3 mice (m1 = 32 barcodes, m2 = 17 barcodes, m3 = 11 barcodes).

Previously, lentiviral barcoding analysis has shown that lympho-myeloid-primed progenitors (LMPPs) include progenitors only giving rise to cDCs, suggesting that commitment to the cDC lineage can occur very early in hematopoiesis (18). In addition, some LMPPs were giving rise to only one of the cDC1, cDC2 and plasmacytoid splenic DCs subsets while very few LMPPs were producing all DC splenic subtypes. In mice, LMPPs are a subset of a broader hematopoietic stem and progenitor cell population known as multipotent progenitor 4 (MPP4) (39) but it is not known if results from Naik et al. (18) can be extrapolated to all MPP4s, and also if this result holds across all tissues where cDC’s reside. In this analysis, we wish to assess if a single MPP4 can produce both the cDC1 and the cDC2 subsets in the lung or whether a single MPP4 is fate-restricted to only produce cDC1 or cDC2 (Figure 3A).

To address this research question, we used a lentiviral barcoding approach focusing on the differentiation of (Lin^–^, Sca-1^+^, cKit^+^, Flt3^+^) MPP4s towards lung-resident cDCs (Figure 3B). Specifically, MPP4s were purified from the bone marrow of donor mice by fluorescence activated cell sorting and infected with the LG2.2 lentiviral barcoding library (40). Labeled cells where then injected I.V into 3 irradiated recipient mice. Fourteen days later, lungs were isolated from the mice, and barcoded cDC1s, and cDC2s were purified by FACS using the gating strategy shown in Figure 3B. Samples were then processed for barcode detection in genomic DNA by deep sequencing as described in the Materials and Methods section.

### Data quality control

**FIGURE 4:**
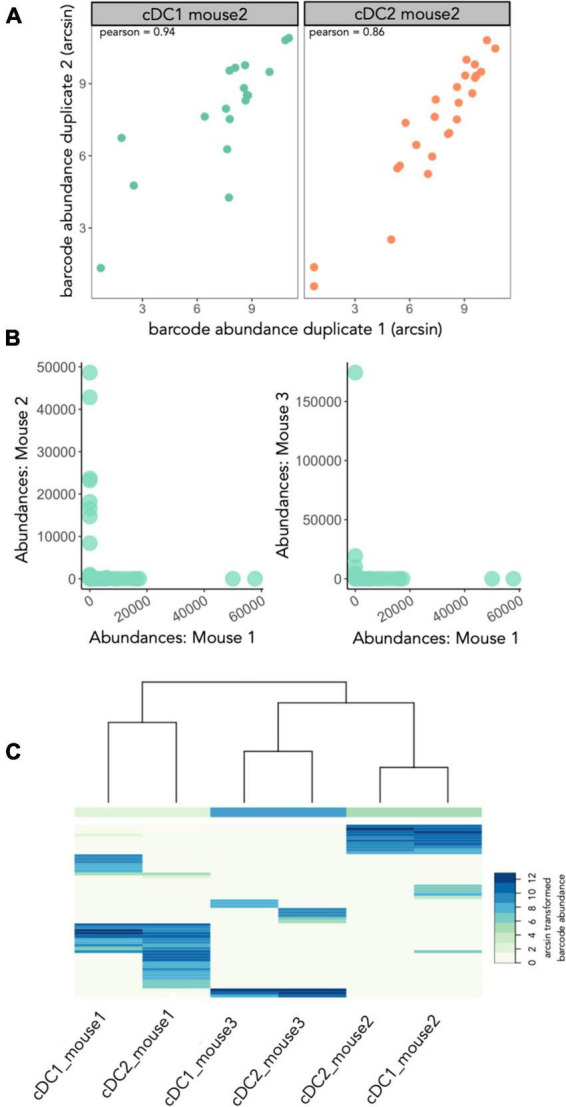
Analyzing the impact of sequencing errors and repeat use barcodes **(A)** comparing technical replicates for cDC1 and cDC2 samples of mouse 2 from case study 1 to control for sequencing errors On these plots each dot represents a single barcode and the Spearmans correlation coefficient is given at the top of each plot (0.79 for the cDC1 sample and 0.93 for the cDC2 sample). This is a representative plot showing data from 1 mouse, figures for the other mice can be found in Supplementary Figure 1. **(B)** Assessing repeat usage of barcodes by comparing barcode abundances across distinct individual mice from case study 1. **(C)** Same as panel **(B)** but using a heatmap to compare all the mice together. Renormalized data is arcsin transformed, clustering based on Euclidian distance and complete linkage. The frequency of repeat use barcodes is 3.4% in this dataset. After sample QC a total of 60 barcodes were recovered for lung cDCs across three mice (m1 = 32 barcodes, m2 = 17 barcodes, m3 = 11 barcodes).

Prior to drawing biological insights from a lineage tracing experiment, it is important to assess whether there are technical factors that can confound the interpretation of the data (34). Before CellDestiny, the samples have been filtered as described in the material and methods giving a total of 60 barcodes represented in the cDC lineages for all 3 mice (m1 = 32 barcodes, m2 = 17 barcodes, m3 = 11 barcodes). Applying the CellDestiny workflow to our case-study lentiviral barcoding dataset, we see a high correlation between technical replicates (example for mouse 2 in Figure 4A) (Spearman’s ρ = 0.85 ± 0.08, *p*-value = 0.01 ± 0.02). By plotting repeat usage diagnostic plots (Figures 4B,C), very few shared barcodes (3.4%) appear across multiple individuals (Figure 4C). Thus, the QC visualization of CellDestiny confirms that our data are of good quality for biological analysis. In the subsequent sections we use CellDestiny to assess clonal diversity and clone-size distributions, as well as lineage bias parameters to study the developmental history of lung-resident cDCs.

### Data exploration and analysis

#### Clonal diversity and clone size in cDC1 and cDC2

To study the ontogeny of differentiated cell types, CellDestiny provides functionalities to assess: (i) clone size distributions and (ii) clonal diversity. CellDestiny provides visualization of how many cells are produced per single barcoded progenitor, here referred to as clone size. Clone size analysis allows to quantify the cellular outputs of barcoded progenitors. In addition to clone size, clonal diversity measures the number of progenitors that give rise to a differentiated cell population and so gives an indication of the amount of active progenitors contributing to differentiation. There are different ways to estimate clonal diversity using CellDestiny, the simplest being the total number of unique barcodes that are measured per sample, however, more complex metrics such as Shannon and Simpson indices that consider how frequently each barcode is measured are also available. These more complex metrics are useful for barcoding data where clone sizes vary significantly amongst different cell types.

**FIGURE 5:**
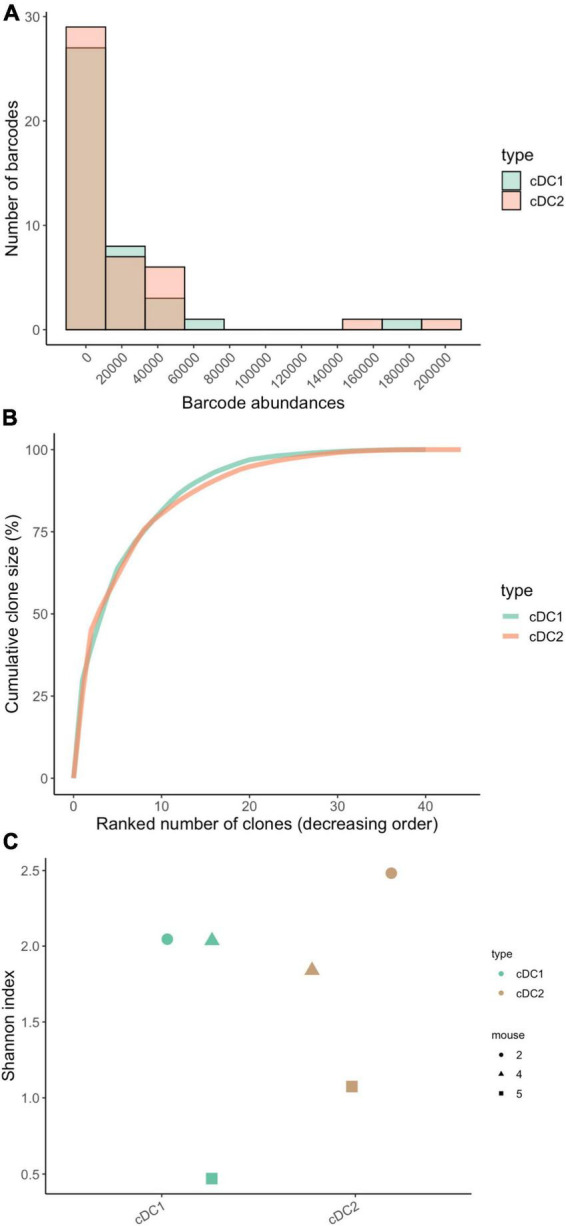
Clone size distributions and clonal diversity between lung-resident cDC1 and cDC2. **(A)** Clone size histograms highlight a similar distribution for both subtypes. **(B)** Cumulative clone size diagram for cDC1 and cDC2. The concave shape highlights a clonal composition made by few relatively big clones with five clones accounting for 60% of the total barcode abundance. **(C)** Dotplots of the Shannon diversity index within each subtype. Each dot represents a single mouse. No statistical difference was observed between the two subtypes (tested with paired-Wilcoxon test, *p*-value = 0.7). *N* = 3 mice for all panels, total of 60 barcodes (m1 = 32 barcodes, m2 = 17 barcodes, m3 = 11 barcodes).

To assess clone sizes of the cDC1 and cDC2 cell types in the lung, we plotted their barcode abundance distributions and observed that not all MPP4 produced the same amount of cDC1 and cDC2 (Figures 5A,B) but that MPP4 had very similar clone-size distributions for both cell types with very few MPP4 (20 barcodes) giving rise to 96% of cDC1 and cDC2 in the lung (Figure 5B).To assess the clonal diversity of the cDC1 and cDC2 we use the Shannon diversity index and found no statistically significant difference (*p* = 0.7) between the cDC1 and cDC2 cell types (Figure 5C). This suggests that a similar number of progenitors is contributing to the production of these subsets although care should be taken when interpreting this result as our sample size is small (*n* = 3 mice, 60 unique cDC-associated barcodes across all three samples) affecting the statistical power of our analyses.

To summarize, we observed no differences in clone size distributions or clonal diversity between cDC1 and cDC2, suggesting that they have similar developmental properties. We find that only a small subset of MPP4s will give rise to the majority of mature cDC1 and cDC2 in the lung. In the following section we will assess whether a single MPP4 can produce both cDC1 and cDC2 by quantifying the proportion of barcodes that are shared across both cell types using CellDestiny.

#### Barcode sharing between cDC1 and cDC2

**FIGURE 6:**
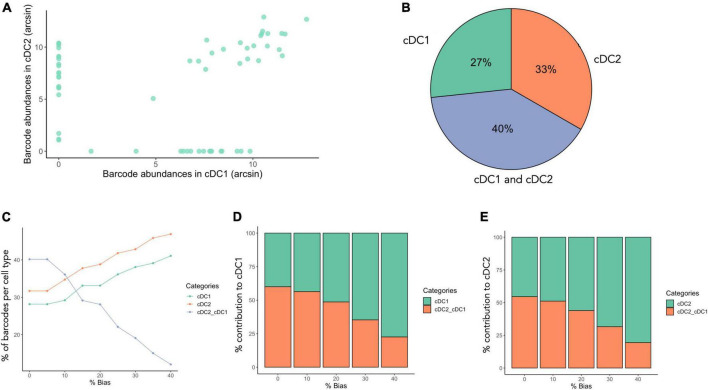
Barcode sharing and lineage bias across lung-resident cDC subsets. **(A)** The dotplot shows the arcsin transformed barcode abundance across cDC1 and cDC2. On this visualization each dot is a distinct barcode. Shared bi-outcome barcodes are on the diagonal and uni-outcome barcodes are on the respective X and Y axes. **(B)** Proportion of total unique barcodes that are shared between cDC1 and cDC2. Bi-outcome barcodes are shown in blue while the red and green segments represent unipotent barcodes for cDC1 and cDC2, respectively. This plot represents data pooled across all three mice. **(C–E)** Lineage bias classifier using different thresholds (indicated on top of the graph, 0–40%) using data pooled from all three mice. *N* = 3 mice, total of 60 barcodes (m1 = 32 barcodes, m2 = 17 barcodes, m3 = 11 barcodes).

To understand if a single MPP4 can give rise to both cDC1 and cDC2, we assess barcode sharing across both subsets (Figure 6A). Proportionally, 40% of barcodes (SD = 16%)were found in both cell types while of the remaining 60%, 28% (SD = 6.7%) of progenitors give rise to only cDC1 subtype, and 32% (SD = 22.4%) to only cDC2 subtypes (Figure 6B). While the majority of clones were uni-outcome, bi-outcome progenitors had larger clone size distributions, suggesting that the majority of cDC1s and cDC2s are derived from a subset of high-output bi-outcome MPP4s. However, a barcoded MPP4 producing both cDC1 and cDC2 did not always produce the same amount of each cell type. For example, an MPP4 that gave rise to 20 cDC1 and 5,000 cDC2 is accounted as bi-outcome if one considers cellular output in binary terms, but this MPP4 may also could also be considered as cDC2-biased due to its unbalanced cellular output. To assess lineage-bias, CellDestiny provides a threshold-based classifier (18, 19) to quantify lineage bias. In this approach, we first calculate the proportional read abundance of each barcode in each cell type. A barcode is then considered biased if the proportional read abundance within a given cell type exceeds a pre-defined threshold value. A barcode is considered multi-outcome if the proportional read abundance exceeds the threshold value across more than one cell type. We applied this classifier to MPP4s using thresholds ranging from 0 to 40% (Figures 6C–E). For bias thresholds ranging from 0 to 20% we found that >50% of progenitors were biased in their production of cDCs.

In summary, CellDestiny was used to assess the ontogeny of lung-resident cDCs. While clonal diversity and clone size distributions were not significantly different for cDC1 and cDC2, we find that the MPP4 population is functionally heterogeneous with respect to lung DC production, containing both progenitors producing one of the DC subtype or both, as well as lineage-biased progenitors. This suggests that fate commitment to the lung cDC cell types can occur early in differentiation already at the MPP4 stage. In the following section, we demonstrate the versatility of CellDestiny to analyze other types of lineage-tracing data, focusing on integration site analysis of patients undergoing gene-therapy.

## Case study 2: Integration site analysis of gene-therapy patients

### Overview

Wiskott-Aldrich Syndrome (WAS) is a monogenic X-linked primary immunodeficiency characterized by thrombocytopenia, eczema, bleeding episodes, and immunodeficiency (41). The disorder is caused by mutations in the WAS gene, which codes for WASP, a protein that regulates the cell cytoskeleton. A 2013 study by Aiuti et Al. assessed the dynamics and efficacy of gene-therapy treatment in three WAS patients through longitudinal lentiviral integration site analysis (30). In this setting autologous CD34^+^ hematopoietic stem and progenitor cells (HSPCs) harvested from patient bone marrows were transduced with a functional WAS gene *ex vivo* before being reinfused intravenously into patients. In transduced HSPCs, lentiviruses randomly integrate into the genome. The genomic coordinates of the lentiviral integrations are unique to each transduced cell and are inherited by the cell’s progeny. The integration sites thus act as heritable markers that can be used to assess clonal dynamics of transduced HSPCs in these patients. Here we use CellDestiny to explore clonal diversity, clone size distributions and lineage commitment of transduced HSPCs in WAS patients. We started our analysis with the filtered matrix kindly provided by the authors of the paper (30) and show how some of the authors conclusions (30) can be independently verified using CellDestiny.

### Clonal dynamics in Wiskott-Aldrich Syndrome patients undergoing gene therapy

**FIGURE 7:**
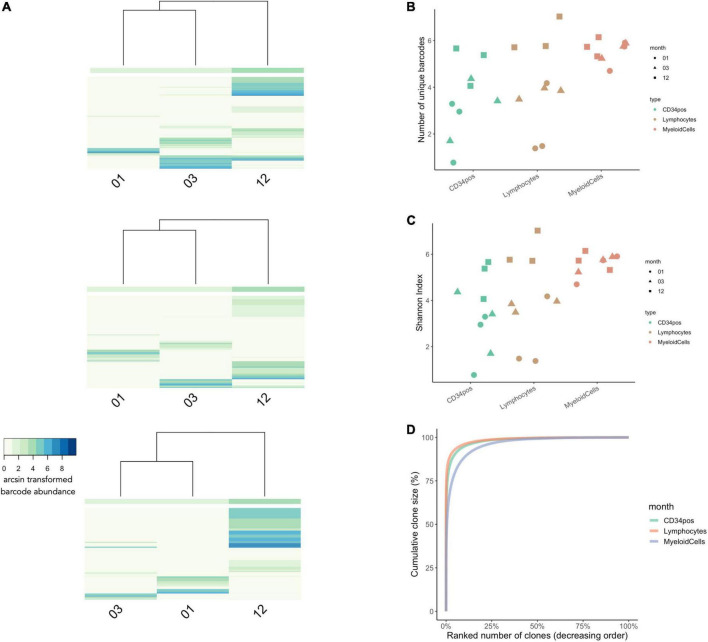
Clonal Diversity in WAS Patients Undergoing Gene-Therapy from 1 to 12 months post-transplantation. **(A)** Unsupervised hierarchical clustering and heatmap visualization of barcode abundances across timepoints for each patient. The x-axis shows the timepoint post-transplantation. The y-axis corresponds to each unique IS (patient 1 had a total of 4,397 unique integration sites, patient 2 had a total of 5,317 integration sites, and patient 3 had a total of 6093 integration sites). The colors indicate the arcsin transformed read abundance. Hierarchical clustering was performed using the Euclidian distance and complete linkage. **(B)** The number of unique IS per cell lineage at 1, 3, and 12 months post-transplantation (in green, red, and blue, respectively). **(C)** Comparisons of Shannon Index measures across cell types and timepoints. **(D)** Cumulative clone size diagram shows that 25% of clones made up to 99% of the total IS abundances for all three time points [same colors as in panel **(B)**]. *N* = 3 patients in all graphs.

To understand clonal dynamics shortly after transplantation in patients undergoing gene therapy, we first assess clonal diversity at 1 month, 3 month, and 12 months post-transplantation in bone-marrow CD34^+^ HSPCs and peripheral-blood leukocytes (Figure 7). Unsupervised clustering and heatmap visualization showed relatively few barcodes were observed across more than one timepoint (Figure 7A). This observation could be due to different HSPCs reconstituting the hematopoietic system over time or could be a sampling artifact given that only a small percentage of the total blood volume was sampled at a given timepoint, preventing the analysis of barcode fate over time.

Looking at the number of unique barcodes detected at each timepoint, we observed that clonal diversity across CD34^+^ cells and leukocytes increases over time (Figure 7B). We also observed lineage-specific differences in clonal dynamics, whereby myeloid diversity remained relatively stable across all timepoints while in lymphocytes and CD34^+^ progenitors’ clonal diversity increased at each subsequent timepoint. For lymphoid cells, this is consistent with the slower dynamics of thymic reconstitution. At 12 months, the clonal diversity was higher in lymphoid cells than in the other lineages, consistent with the selective growth advantage effect of the gene corrected clones in this lineage (6). Similar trends were also observed using the Shannon diversity index (Figure 7C). Finally, investigating clone size composition over time, we observe that the 25% of IS clones with the highest rates of cellular output make up to almost 100% of the total abundances at all time points (Figure 7D).

Using CellDestiny, we show that the reconstitution in these WAS patients was carried by an increasing number of HSPC clones during the first 12 months post-transplantation. In the next section, we will analyze cell production from HSPCs to the different lineages over time to assess if reconstitution was performed by multi-outcome or lineage-biased HPSC clones.

### Lineage commitment of transduced hematopoietic stem and progenitor cells

**FIGURE 8:**
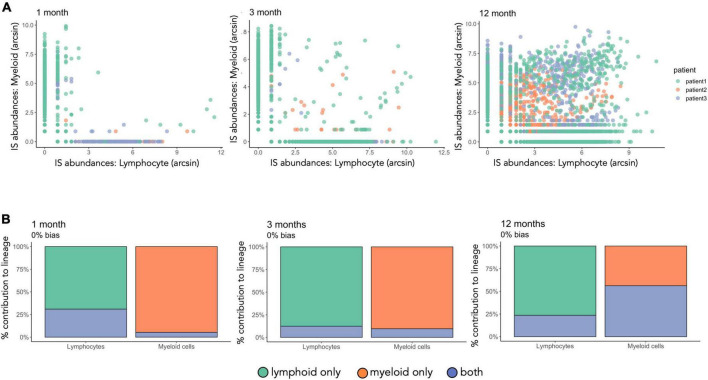
Increased proportion of bi-outcome HSPC from 1 to 12 months after gene therapy. **(A)** IS abundances in lymphocytes versus myeloid cells for each timepoint. Each dot is an IS color-coded by patient. The number of sequencing reads per IS has been renormalized and arcsin transformed. **(B)** IS have been classified by presence or absence in the myeloid and lymphoid cell lineages. The total output of uni-outcome or bi-outcome HSPCs to myeloid cells and lymphocytes is computed per patient. Here we show the mean value over the three patients. Green and orange correspond to IS found only in the lymphoid or myeloid lineage, respectively (uni-outcome), blue corresponds to IS found in both lineages (bi-outcome).

**FIGURE 9:**
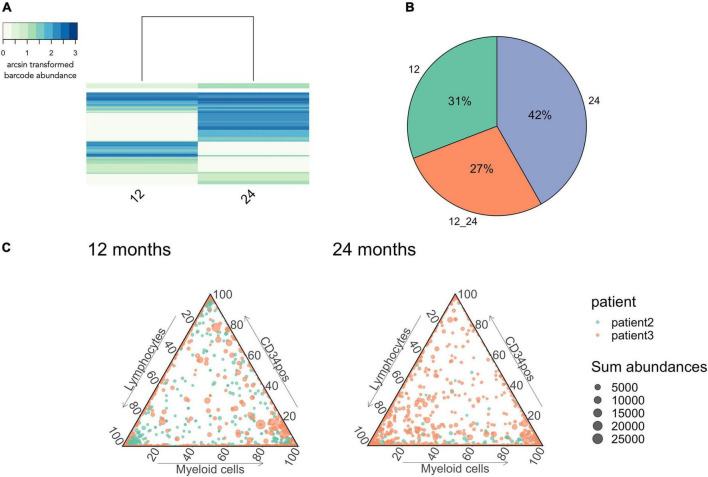
Stabilization of multi-output HSPCs detected at 24 months after gene therapy. **(A)** IS sharing between 12 and 24 months. Heatmap done as in Figure 6. **(B)** Percentage of barcodes shared between lymphocytes and myeloid cells at 24 months. **(C)** IS abundance in myeloid cells, lymphocytes and CD34positive cells. Each dot represents an IS, colored coded per patient while the size of each dot represents the total number of reads over the 3 lineages. *n* = 2 patients with patient 2 having in total 1,429 integration sites and patient 3 2,289.

To understand the cell production of transduced HSPCs in different hematopoietic lineages, we compared IS barcodes across peripheral-blood myeloid and lymphoid immune cells at 1,3,12, and 24 months post-transplantation (Figures 8A, 9). At 1 and 3 months post-transplantation, the majority of IS were not shared between lymphoid and myeloid cells whereas at 12 months more IS are shared between the two lineages (Figure 8A). From 1 to 12 months post-transplantation, bi-outcome HSPC numbers increased from representing 5% at 1 month to 20% of total IS barcodes detected. Bi-outcome HSPC produced 24 and 56% of lymphoid and myeloid cells respectively at 12 months post-transplantation (Figure 8B). Overall, the majority of blood cells were produced by uni-outcome progenitors at each time point. This shows that the first wave of hematopoiesis in WAS patients was sustained primarily by lineage committed progenitors.

Lastly, we observed persistent multi-outcome HSPCs from 12 to 24 months after gene therapy (27%, Figures 9A,B), illustrating that reconstitution is stabilizing in these patient after 12 months post-transplantation. We also observed a large proportion of new clones that had not previously been detected (42%, Figures 9A,B), corresponding to either the emergence of new HSPC clones or a sampling artifact due to the low volume of blood sampled at each timepoint. At 12 and 24 months post-treatment, we also observed increasing numbers of multi-outcome HSPCs (dots at the center of the triangle, Figure 8C). Through the various graphics available in CellDestiny, we have independently verified the major conclusions from the original paper that hematopoietic reconstitution follows a first unstable phase sustained by lineage committed progenitors that from 12 months post-transplantation is slowly replaced by stable reconstitution from multi-outcome HSPCs.

## Case study 3: Combining single-cell RNA sequencing and lineage tracing information in single Cells

### Overview

**FIGURE 10:**
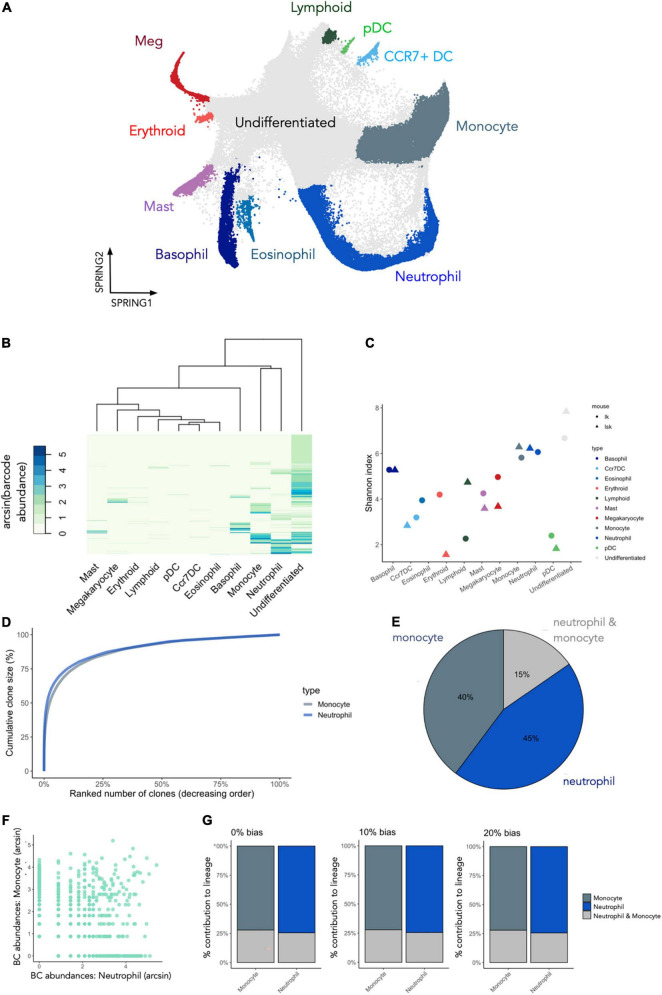
Using CellDestiny to analyze datasets with transcriptomic and cellular barcoding measurements of single cells. **(A)** a UMAP visualization of gene expression data for cKit+ (LK) and cKit+ Sca1+ (LSK) murine bone marrow cells that were barcoded and then differentiated *in vitro*. Each color represents a distinct cell type as designated by the authors of the original study. These cell type references were used to generate a count matrix by tallying the number of times each barcode occurs in each cell type **(B)** Hierarchical clustering of the lineage tracing data using CellDestiny using Euclidian distance and complete linkage. Colors represent the arcsin transformed number of cells per barcode per cell type. **(C)** Shannon diversity measures for all cell types. Each cell-type is represented with two points, with each point representing either the LK or LSK starting cell population **(D)** cumulative clone-size distributions for the monocyte and neutrophil clusters. **(E)** The proportion of barcodes that are unique to, or shared between the monocyte and neutrophil clusters. **(F)** A scatterplot of arcsin transformed barcode abundances for the monocyte and neutrophil clusters for LSK and LK subsets of the data. **(G)** Lineage bias classifier results for barcodes of the neutrophil and monocyte cell types using 0 and 20% thresholds. *N* = 3 experiments, *N* = 5865 barcodes.

Recent technical developments in the lineage tracing field now permit simultaneous fate and gene expression measurements in single cells (42) (Figure 10). To illustrate how CellDestiny can be used to analyze such datasets we present a case study analysis wherein cKit^+^ (LK) and cKit^+^ Sca1^+^ (LSK) murine bone marrow cells were barcoded using the LARRY lentiviral barcoding library and then cultured in vitro using a cocktail of cytokines and growth factors that support pan-myeloid differentiation (Figure 10A) (35). In this published study, cells were processed for single cell RNA sequencing at days 2,4, and 6 after lentiviral barcoding and barcode information was recovered at the RNA level directly from InDrop sequencing libraries along with associated gene expression measurements. To convert this dataset into a format compatible with CellDestiny we generated a count matrix, where count values represent the number of times each barcode was found in each cell type (refer to the materials and methods section for further details). Cell type definitions were taken from the original publication and were obtained by k-means clustering of gene expression measurements and then annotating the clusters using marker genes from the literature (Figure 10A). This matrix was then loaded into CellDestiny along with metadata about each starting cell population (LK and LSK). The aim of this analysis is to visualize barcode abundance and diversity across different cell types as well as visualising barcode clone size distributions, and patterns of barcode sharing between myeloid cell types that are highly represented in this dataset. This important information cannot be obtained using standard single-cell RNA analysis toolkits such as Seurat and scanpy.

### Clonal composition of in vitro differentiated murine progenitors

To visualize barcode expression patterns across all cell types in the dataset we performed hierarchical clustering and heatmap visualization in CellDestiny (Figure 10B). This analysis showed that most barcoded progenitors differentiated into basophils, monocytes and neutrophils in this culture system, a result corroborated by quantifying barcode diversity in each cell type using the Shannon Index (Figure 10C). Diversity measures across differentiated cell types were similar for the myeloid lineages irrespective of whether in vitro cultures were seeded with LK and LSK starting cell populations (Figure 10C). Larger differences were observed when assessing erythroid and lymphoid lineages, however (Figure 10C). The heatmap visualization and barcode diversity plots also showed that a number of progenitors did not share barcodes with any mature differentiated cell types (Figure 10B)—suggesting they may be differentiation inactive–a progenitor phenotype that has also been described *in vivo* using *in situ* barcoding (8, 28, 43). Lastly, we observed that the dominant barcodes for each cell type were typically not observed in the dominant barcodes of other cell types. This suggests that many progenitors in this culture system are lineage-biased (Figure 10B).

### Monocytes and neutrophils lineage commitment *in vitro*

Given that monocytes and neutrophils were the dominant cell types fates in this assay, we focused our analysis of their developmental trajectories. Firstly, both cell types had similar diversity scores, as well as having very similar clone size distributions (Figures 10C,D). This result suggests that similar number of progenitors contributed to each lineage, and the relative number of mature cells produced per progenitor were similar. Despite these similarities there was only a small degree of barcode (15%) sharing between monocytes and neutrophils, suggesting that the majority of progenitors produced only one of the two cell types (Figure 10E). Scatterplot visualization of barcode abundances showed that shared barcodes were not more prevalent that non-shared barcodes (Figure 10F). This increases confidence that lineage restriction cannot be explained by sampling biases whereby clones would appear to be non-shared simply because they are rare and therefore more likely to escape detection. In addition, we observed the same pattern when analysing LSK and LK cells separately, ruling out the hypothesis that this pattern is driven only by Sca1^–^ lineage-restricted progenitors such as GMPs (Figure 10F). We corroborated this result by performing a lineage-bias classification analysis which takes the relative abundance of each barcode in each lineage into account. This analysis showed that the majority of the monocytes or the neutrophils were produced by uni-lineage progenitors, irrespective of the threshold used to classify each progenitors (Figure 10G).

Through the various graphics available in CellDestiny, we have independently re-analyzed a published dataset combining lineage tracing and gene expression analysis of single cells. Our analyses suggest that lineage decisions in this system occur at the level of LSK cells. This is consistent with the conclusions reached in the original study, wherein the authors also incorporated transcriptomic and temporal information to enrich their analyses. Importantly, key lineage tracing metrics such as clonal diversity, clone size distributions and barcode sharing cannot be assessed using existing single cell RNA sequencing toolkits. This case study illustrates how CellDestiny can be used to efficiently visualize and explore complex lineage tracing datasets, identifying key trends at the level of barcode expression patterns to direct and inform informing downstream statistical analyses as well as multi-omics data integration.

## Discussion

To summarize, single-cell lineage tracing is a powerful tool to study cellular genealogies *in vivo*, with many applications for fundamental and applied biomedical research. In recent years, a number of single-cell lineage tracing methodologies have emerged, but tools to analyze such data remain limited. This limitation is critical as lineage tracing datasets are complex and multidimensional, characterized by high levels of variability, sparsity and technical noise. Context-specific bioinformatics pipelines have been developed to address these challenges, but the lack of standardized approaches makes it difficult to compare results across independent studies. This issue is further exacerbated by the technical demands of lineage tracing, with very few researchers capable of performing both experiments and data analysis steps. User-friendly analytical software is important for increasing the accessibility and reproducibility of lineage-tracing, and for bridging the gap between specialists in data generation and specialists in data analysis.

In this article we present an open-source computational toolkit, CellDestiny, that performs visualization and analysis of lineage-tracing datasets, complementing the existing genBaRcode and barcodeTrackR which focus on barcode pre-processing and longitudinal analyses, respectively. All three toolkits address the fundamental challenge of making single cell lineage-tracing analysis more accessible and reproducible, while the specific advantages of CellDestiny are: (i) the ability to incorporate study metadata into the analysis framework permitting greater versatility, (ii) the focus on visualizing experiment-level quality control metrics, providing visualization tools to assess the consistency of technical replicates, and to assess the frequency of repeat used barcodes. Thus, prior to drawing biological conclusions from the data, the user can assess whether or not critical data pre-processing steps have been performed correctly. (iii) Analysis versatility, in that CellDestiny allows the user to choose which data to visualize using several grouping options, for example the ability to assess clone sizes and clonal diversity across individuals, organs and cell types.

The purpose of this app is to make lineage tracing analysis more accessible to people who do not have a background in computer programming. We anticipate that this can improve the efficiency of interdisciplinary teamwork where in our experience a lot of time can be wasted in generating plots that end up not being useful because nuanced details about the experimental design and implementation were not considered, or in adjusting existing plots to better emphasize key results. Importantly, this app is intended as an exploratory tool, and is not intended to replace rigorous statistical analysis and testing of the data which should be performed by data analysis specialists. As a concrete example we may observe a statistically significant difference in barcode diversity between two individuals, but such an effect could potentially be explained by sampling biases. We therefore strongly advocate that biological insights obtained by using the app should be independently assessed through rigorous statistical analysis.

Through case studies in lentiviral barcoding and lentiviral integration site analysis we illustrate the functionality and versatility of CellDestiny. Specifically, we show how CellDestiny can be used to assess data quality, as well as analyzing clonal diversity, clone-size distributions and lineage commitment parameters. Lentiviral barcoding analysis of murine hematopoietic progenitors leads us to conclude that commitment to either the lung-resident cDC1 or cDC2 fate can occur in the bone marrow, consistent with a previous barcoding study (18). Our data brings new insights into this topic as Naik et al. (18) studied LMPPs, which represent only a subset of the entire MPP4 population, and did not study the development of lung-resident cDCs. This result has important ramifications for our understanding of immune cell development, challenging previous studies suggesting that cDC fate-commitment was thought to occur at later stages of hematopoiesis (44). We complement this analysis with an additional case studies in lentiviral integration site analysis and paired lineage tracing and single cell transcriptomics measurements, showing how CellDestiny can be used to analyze different types of single cell lineage tracing data. As long as datasets are available in a count matrix format where rows are cell identifiers (barcode sequence, insertion site, etc.) and columns are samples, they are compatible with CellDestiny, for example CRISPR-Cas9 barcoding (8), polylox barcoding (12), and single-cell RNA barcoding (35).

To summarize, CellDestiny is an easy to use and versatile open-source software toolbox that permits the visualization and analysis of lineage tracing data. We anticipate that CellDestiny will play a key role within a broader ecosystem of analysis tools that increase the accessibility, reproducibility and standardization of single cell lineage-tracing approaches with implications for basic research and for gene therapies.

## Materials and methods

### CellDestiny

#### CellDestiny format

CellDestiny is designed for biologists without coding skills and is available as a Rshiny app and through a web-interface. We also make available the package – all of which can be accessed here:

R package: https://github.com/TeamPerie/CellDestiny.

Web application: https://perie-team.shinyapps.io/CellDestiny/

#### Input data format

Lineage tracing dataset are typically organized in a matrix of absolute read counts where rows are cell identifiers (barcode sequence, insertion site, etc.) and columns are samples and so this is the input format of CellDestiny. Two types of data matrix can be loaded to CellDestiny. The first matrix is for data quality control which requires a matrix with technical replicates across individuals (e.g., sample1_dupA, sample1_dupB) to check for PCR errors and repeat usage. CellDestiny also accepts as input count matrices where technical replicates are merged (e.g., by summing values or taking the mean value across technical replicates).

The second type of input data is a metadata file giving Supplementary Information about study design and sample information for instance cell types, organs, treatments, etc. An example metadata input file can be found at: https://github.com/TeamPerie/HadjAbed-et-al._2022.

#### Data preprocessing

A pre-processed count matrix can be loaded in the QC module of the application to visualize key quality control metrics (Figure 1). In the CellDestiny package, the functions ReformatQCmatrix(), MakeDuplicatesMatrix() and MakeRepeatUseMatrix() create the input matrices to check QC by plotting the barcode sharing between duplicates using the function PlotDuplicates() and between individuals using the function PlotRepeatUse(). Once these QC metrics have been visualized read counts from duplicates are summed or averaged resulting giving a new matrix that can be loaded in the analysis module of the application.

#### Barcode sharing

To explore barcode sharing across samples, CellDestiny allows the user to visualize shared barcodes between two cell types cell types (using the MakeDotplotMatrix() and PlotDotplot() function in the package). In the dotplot visualization, barcode abundances can be plotted on different scales (logarithmic or arcsin). The dotplot can be complemented by a piechart (using MakePieChartMatrix() and PlotPieChart() in the package) which gives the percentage of shared and unshared barcodes between the two variables of choice. Shared barcodes can also be assessed across 3 cell types cell types with ternary dotplots (using the MakeTernaryPlotMatrix() and PlotTernaryPlot() function in the package). In the ternary plot, dot size varies with the total abundance of the barcodes in the samples. Alternatively, barcode sharing between more than three cell types can be visualized as a heatmap.

#### Lineage bias

To classify barcodes by their lineage bias, CellDestiny uses a threshold based classifier lineage described (18). In summary, an additional normalization step per barcode is applied in each individual, thereby enabling categorization of each barcode into classes of biased output towards the analyzed cell types. Specifically, in our absolute count matrix where rows are barcodes and columns are samples, we first normalize the absolute read counts by the sample specific total number of reads (column-wise normalization). Following this step we then normalize each barcode such that each row sums to 1 (row-wise normalization). This allows us to see how the barcode is distributed across different cell types.

Barcodes are assigned a bias based on whether the % read abundance exceeds a threshold value. If one barcode contributes to a given lineage above the designated threshold then this barcode is assigned to be biased towards that lineage. Barcodes for which the % read abundance exceeds a threshold value across multiple lineages are classified as multi-outcome. More precisely:

Let R_bc_ represent the number of reads for barcode *B* in cell type *C*, and let P_bc_ represent the proportional read abundance per barcode per cell type


$$P_{bc}=\frac{R_{b\,c}}{\sum_{i}\;R_{bc}}$$

The barcode is then classified as lineage biased if P_*bc*_ equals or exceeds a threshold value


$$B=\left\{ \begin{array}{c} \;u\,n\,b\,i\,a\,s\,e\,d,P_{bc}<t\,h\,r\,e\,s\,h\,o\,l\,d\\ b\,i\,a\,s\,e\,d,P_{bc}\geq t\,h\,r\,e\,s\,h\,o\,l\,d \end{array}\right.$$

In the CellDestiny app, the threshold used for categorization can be tuned manually. In the CellDestiny package, MakeCategoryMatrices() prepare the data to input the PlotCategories() and the complementary PlotCategoryCounts() functions that output the number of barcodes per category and the summed contribution of all the barcodes in this category. If several individuals are present, PlotCategoryCounts() averages the summed contribution over individuals.

#### Heatmap and correlogram

Similarities between samples can be visualized using a heatmap together with hierarchical clustering. In the package, the function MakeHeatmapMatrix() prepare the data to plot heatmaps using the PlotHeatmap() function. Several options are available for the distance and algorithm used for clustering. Additionally, a correlogram can be plotted (MakeHeatmapMatrix() and PlotCorrelogram() in the package) that illustrates the correlation of barcode abundances between all pairs of variables.

#### Clone size

CellDestiny offers two types of clone size visualizations. The first one is a cumulative diagram (use MakeCumulativeDiagramMatrix() and PlotCumulativeDiagram() in the package). If the cumulative graph has a concave shape, it means that a cell population is dominated by a small number of large clones. On the contrary, if the shape is linear, the sample is composed of a number of clones which contribute equally to the cellularity of the population. The second type of graph is a frequency distribution plot (MakeBarcodeFrequenciesMatrix() and PlotBarcodeFrequencies ()) in the package) where the user can choose between histogram or density curve -based representations of the data.

#### Diversity

Comparing sample diversities is a common step in lineage tracing analysis. Diversity is computed using the vegan R package. In the CellDestiny package, the function CalculDiversity() calculate diversity using the number of unique clones, the Shannon index or the Simpson index. For vizualizing clonal diversity, PlotDiversity() computes a boxplot of barcode diversities.

### Integration site data

The data used in this article comes from reference (30). The authors of the article provided us the data for two patients over 24 months and for 3 patients over 12 months. The data were already filtered and samples were renormalized to 10^5^.

### Lentiviral barcoding data

#### Mice

Male C57BL/6J CD45.1^+^ and C57BL/6J CD45.2 mice were ordered from Jackson Laboratory or bred at Institute Curie. Mice aged between 7 and 13 weeks were used in all experiments. All procedures were approved by the responsible national ethics committee (APAFIS#10955-201708171446318 v1).

#### Barcode library, barcode reference list

The barcode library used consists of 98 bp semi-random DNA fragments in the 3’ UTR of a GFP cassette as described by (45) and described in detail elsewhere (46). The barcode reference list is available on https://github.com/TeamPerie/HadjAbed-et-al._2022.

#### MPP4 isolation, transduction, and transplantation

Isolation and labeling of cells with the barcoding library was performed as previously described (18). Briefly, after isofluorane anesthesia and cervical dislocation, bone marrow cells were isolated from femur, tibia and iliac bones of 2 donor mice per 4 recipient mice by flushing, and c-Kit^+^ cells were enriched by MACS with anti-CD117 magnetic beads (Miltenyi). Cells were stained for antibodies against CD117, CD135, CD150, Sca-1 and MPP4 were sorted using a FACSAria (BD Biosciences) IIIu as CD117^+^/Sca^–^1^+^/CD150^–^Cd135^+^ (see Figure 2) and transduced with the barcode library in StemSpanMedium SFEM (STEMCELL Technologies) with 50 ng/ml mSCF (STEMCELL Technologies) through 1.5 h of centrifugation at 300 *g* and 4.5 h incubation at 37°C to obtain ∼10% barcoded cells. After incubation, the cells were transplanted by tail vein injection into recipient mice that were 6 Gy sub-lethally irradiated using an X-ray generator 3 h prior to the transplantation.

#### cDC isolation

Mice were euthanized 14 days after transplantation by cervical dislocation. Lung were flushed by transection of the caudal vena cava followed by injection of 2 ml PBS into the right ventricle. Lungs were excised and perfused with digestion medium (HBSS supplemented with 13 U/ml LiberaseTM and 10 mg/ml DNAse I) by injecting medium in each lung lobe until each lobe was fully inflated. Lungs and remaining digestion medium were placed into GenteMACS C tubes. A single-cell suspension was obtained by first incubating the lungs for 15 min at 37°C and subsequently placing them on a GentleMACS Octo dissociator running the 37C_m_LDK_1 protocol at 37°C according to manufacturers’ instructions. The resulting single-cell suspension was filtered using a 100 um cell strainer and MACS enriched for CD11c+ cells using CD11c UltraPure Microbeads (Miltenyi Biotec) according to manufacturers’ instructions. FACS staining was performed on CD11c-enriched single-cell suspensions by first staining dead cells using Live/DEAD aqua in PBS, followed by 30 min antibody staining with CD45.2 Pacific blue, CD11c APC, MHC-II APC-eFluor780, CD3e BV510, CD19 BV510, NK1.1 BV510, Ly6G BV510, CD24 PE, CD103 PE-CF594 and CD11b PerCP-Cy5.5 in PBS supplemented with 1% FBS. Single-cell suspensions were washed and fixed using 1% PFA for 15 min. Within 24 h after antibody staining GFP+/CD45.2+ cDC were sorted (see Figure 2) using a BD Aria IIIu. After sorting, cells were lysed in Viagen Direct PCR lysis Reagent supplemented with 0.5 mg/ml Proteinase K at 55°C for 2 h and 85°C after which Proteinase K was inactivated at 95°C for 5min. Lysed cells were frozen at –20°C until further processing.

#### Barcode amplification and sequencing

Barcodes were amplified as published previously (46). In short, lysed cell preparations were split to allow for duplicate measurements. Barcodes in the preparations were first amplified by PCR using forward tgctgccgtcaactagaaca and reverse gatctcgaatcaggcgctta primers. Subsequently, a second amplification step was performed using the sample primers, but incorporating Illumina P5 and P7 flow cell sequences and sample index primers. PCR products for sample replicates pooled, purified with the Agencourt AMPure XP system (Beckman Coulter), diluted to 5 nM. and sequenced on a HiSeq system (Illumina) (SR-65bp) at Institute Curie facility with 10% of PhiX spike-in.

#### Barcode sequence analysis

Sequencing results were filtered as in reference (18) and further explained on Github.^1^ In brief, sequencing results were analyzed using R-4.0.3 (R Development Core Team (47)),^2^ Excel, and GraphPad Prism version 8.0 for Mac (GraphPad Software, La Jolla, CA, USA).^3^ Reads were first filtered for perfect match to the input index- and common-sequences using XCALIBR^4^ and filtered against the barcode reference list. Samples were then normalized by dividing the number of reads per barcode per sample by the total number of reads per sample. For filtering, all samples had a Pearson correlation between duplicates higher than 0.8 and were kept. In addition, barcodes present in one of the two replicates were set to zero. After filtering, read counts from duplicates are summed and renormalized. For heatmap plotting this value was then scaled by multiplying the column normalized read counts by 10^5^.

### Analysis of simultaneous transcriptomic and lineage tracing measurements in single cells

Gene expression matrices and associated metadata from Weinreb et al. (35) were downloaded from https://github.com/AllonKleinLab/paper-data/tree/master/Lineage_tracing_on_transcriptional_landscapes_links_state_to_fate_during_differentiation. This dataset comprises 130887 cells, 13920 unique genes, and 5865 unique barcodes. To convert this data into a count matrix compatible with CellDestiny, cells were grouped under the ‘cell type annotation’ descriptor in the project metadata and then for each cell type we calculated the number of cells expressing each barcode. No additional QC processing or normalization steps were performed on the data.

## Data availability statement

The datasets presented in this study can be found at https://github.com/TeamPerie/HadjAbed-et-al._2022.

## Ethics statement

All procedures were approved by the responsible national ethics committee (APAFIS#10955-20170817144 6318 v1).

## Author contributions

LH-A designed and developed the software and performed data-analysis. TT performed wet-lab experiments and data analysis. JC analyzed and interpreted results and wrote the manuscript. LP designed experiments, analyzed and interpreted results, coordinated the research, and wrote the manuscript. All authors contributed to the article and approved the submitted version.

## Footnotes

1
https://github.com/TeamPerie/HadjAbed-et-al._2022

2
http://www.R-project.org

3
www.graphpad.com

4
https://github.com/NKI-GCF/xcalibr
