# Supplementary material for: CellDestiny: A RShiny application for the visualization and analysis of single-cell lineage tracing data
Source: Front Med (Lausanne). 2022 Oct 5;9:919345. doi: 10.3389/fmed.2022.919345 (PMC9581332; doi:10.3389/fmed.2022.919345)
Supplement: Supplementary file 1 [file Data_Sheet_1.pdf]

frontiers

# *Supplementary Material*

| | CellDestiny | barcodeTrackR |
|---|---|---|
| **Inputs** | | |
| Count Matrix | Yes | Yes |
| Metadata | Variable list (celltypes, organs, etc.) | Sample list |
| **Experimental Design** | | |
| Data Type | Longitudinal and Cross-Sectional Data | Focus on Longitudinal Data |
| **Data QC** | | |
| Technical Replicates | Yes | No |
| Repeat Usage of Barcodes Across Independent Samples | Yes | No |
| **Data analysis** | | |
| Lineage Bias Classifier (Categorisation) | Yes | No |

| | | |
|---|---|---|
| Clone Sizes | Yes | Yes |
| Barcode Sharing | Yes | Yes |
| Correlation analysis | Yes | Yes |
| Heatmap | Yes | Yes |
| Diversity | Yes | Yes |
| Group plotting variables by metadata | Yes | Only for a small subset of plots |
| **Outputs** | | |
| Data matrix | Yes | Yes |
| Visualization Plots | Yes | Yes |
| **Platform** | | |
| RShiny package | Yes | Yes |
| RShiny Web application | Yes | Yes |

**Table S1** Comparison of the CellDestiny and barcodeTrackR packages on different aspects of their use.

| Case Study | Individual | Number of barcodes |
|---|---|---|
| Lentiviral barcoding of hematopoietic progenitors | Mouse 1 | 32 |
| Lentiviral barcoding of hematopoietic progenitors | Mouse 2 | 17 |
| Lentiviral barcoding of hematopoietic progenitors | Mouse 3 | 11 |
| Integration site analysis of patients undergoing gene therapy | Patient 1 | 4397 |
| Integration site analysis of patients undergoing gene therapy | Patient 2 | 5317 |
| Integration site analysis of patients undergoing gene therapy | Patient 3 | 6093 |
| Transcriptomic and fate analysis of murine hematopoietic progenitors *in vitro* | LK | 2186 |
| Transcriptomic and fate analysis of murine hematopoietic progenitors *in vitro* | LSK | 3720 |

**Table S2** Number of barcodes/integration sites per experiment, per individual.

| **Term** | **Definition** |
|---|---|
| *Reference Library* | A list of genetic barcode sequences that is used to distinguish true barcodes from spurious barcodes that arise due to PCR and sequencing errors. |
| *Invariable sequences* | Within most if not all barcoding strategies, each genetic barcode has variable and invariable nucleotide sequences. The invariable sequences are the same across all barcodes. It is used to amplify all barcodes before sequencing and for filtering barcodes. |
| *PCR replicates* | A single sample is split into two technical replicates prior to PCR amplification. Sequences that are found in both replicates are more likely to be true barcodes, and not spurious barcodes that arise due to sequencing errors. |
| *Sampling Replicates* | Within many experimental designs, it is not feasible to sample entire tissues. To quantify the aleatory uncertainty associated with sampling, two samples of the same size can be taken from the same tissue and the consistency of results can be compared. If results are very different between sampling replicates this suggests that the sampling size is too small for the experiment. |

| | |
|---|---|
| *Repeat-Use Barcodes* | When cells from the transduction batch are transplanted into different individuals, the frequency at which we find the same barcode across different individuals is called repeat-use barcode frequency. This can be used to estimate the likelihood of two independent cells being labelled with the same barcode. |
| *Clonal Diversity* | The number of labelled barcoded ancestor cells that give rise to a differentiated cell population. |
| *Clone-Size Distributions* | The cellular outputs of barcoded progenitors, ie the number of cells produced per barcoded progenitor. |

**Table S3** Glossary of technical terms used in the study

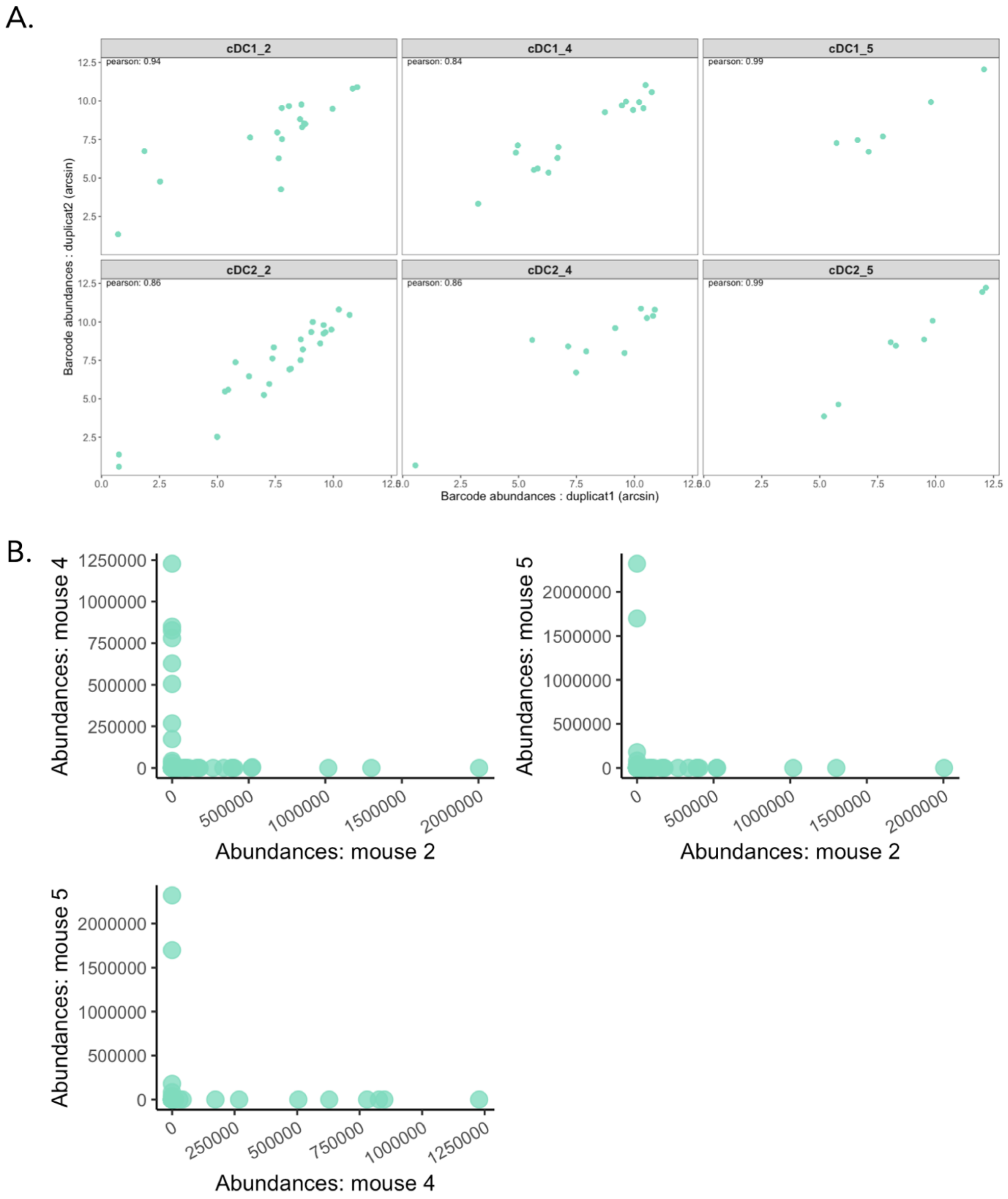


**Figure S1. Experiment level QC metrics for case study 1.** Each point represents a distinct barcode sequence and inset values represent the Pearson correlation between technical replicates showing their correlations. (A) comparing technical replicates for cDC1 and cDC2. Normalised data is arcsin transformed. (B) Assessing repeat usage of barcodes by comparing barcode abundances across distinct individual mice from case study 1. The frequency of repeat use barcodes is 3.4% in this dataset. After sample QC a total of 60 barcodes were recovered for lung cDCs across 3 mice (m1 = 32 barcodes, m2 = 17 barcodes, m3 = 11 barcodes).
